# Providers' Perceptions of Challenges in Obstetrical Care for Somali Women

**DOI:** 10.1155/2013/149640

**Published:** 2013-10-07

**Authors:** Jalana N. Lazar, Crista E. Johnson-Agbakwu, Olga I. Davis, Michele P.-L. Shipp

**Affiliations:** ^1^Lifestages Samaritan Centers for Women, 2200 Philadelphia Drive, Suite 101, Dayton, OH 45406, USA; ^2^School of Social Work, Southwest Interdisciplinary Research Center (SIRC), College of Public Programs, Arizona State University, 411 North Central Avenue, Suite 720, MC 4320, Phoenix, AZ 85004, USA; ^3^Hugh Downs School of Human Communication, Principal Investigator, Community Engagement/Outreach Core (CEOC), Southwest Interdisciplinary Research Center (SIRC), College of Public Programs, Arizona State University, 411 North Central Avenue, Suite 720, MC 4320, Phoenix, AZ 85004, USA; ^4^College of Health Sciences, Walden University, 100 Washington Avenue South, Minneapolis, MN 55401, USA

## Abstract

*Background*. This pilot study explored health care providers' perceptions of barriers to providing health care services to Somali refugee women. The specific aim was to obtain information about providers' experiences, training, practices and attitudes surrounding the prenatal care, delivery, and management of women with Female Genital Cutting (FGC). *Methods*. Individual semi-structured interviews were conducted with 14 obstetricians/gynecologists and nurse midwives in Columbus, Ohio. *Results*. While providers did not perceive FGC as a significant barrier in itself, they noted considerable challenges in communicating with their Somali patients and the lack of formal training or protocols guiding the management of circumcised women. Providers expressed frustration with what they perceived as Somali patients' resistance to obstetrical interventions and disappointment with a perception of mistrust from patients and their families. *Conclusion*. Improving the clinical encounter for both patients and providers entails establishing effective dialogue, enhancing clinical and cultural training of providers, improving health literacy, and developing trust through community engagement.

## 1. Introduction

Somalis represent the largest influx of African refugees to the United States of America (USA) [1]. They began arriving in the early 1990s when their country became engulfed in armed conflicts and its citizens were forced to flee for survival [1–3]. Repeated humanitarian crises in Somalia contributed to the continual exodus of millions of Somalis throughout the years. The 2011 report from the US Department of Homeland Security [4] identifies Somalia as one of the leading African countries for refugee admissions in the USA. In Ohio, the Somali Community Association of Ohio (SCAO) estimated that more than 45,000 Somali refugees/immigrants had resettled in Columbus, Ohio, in 2010, with an estimated 200 arriving each month due to both direct resettlement and secondary migration [5, 6]. Columbus and Franklin county officials projected this number to be closer to 80,000, which would represent the second largest population of Somalis in the USA (behind Minnesota) and would account for 42% of all immigrants in Ohio [1, 7]. This increasing demographic diversity among Columbus residents has a profound impact on local population health profiles and health care needs. Somali immigrants form a very homogeneous population linguistically, religiously, and culturally. According to the Ohio Department of Public Safety [8], 99.9% of Somalis in Columbus are Sunni Muslims and share the same ethnic background (except for a minority Bantu group). They are also linked by similar experiences of violence, trauma, loss, food insecurity, and grief. An important shared cultural factor among Somali women is the traditional practice of Female Genital Cutting (FGC), otherwise known as Female Circumcision (FC) or Female Genital Mutilation (FGM), which consists of varying degrees of excision of tissue involving the periclitoral region, labia minora, and/or majora. Somalis have among the highest prevalence rates of FGC in the world (97.9%) [9]. A prospective study across six African countries has demonstrated a trend towards adverse obstetric and neonatal outcomes with increasing severity of FGC when compared to those without FGC, including cesarean delivery, postpartum hemorrhage, extended maternal hospital stay, resuscitation of the infant, and inpatient perinatal death [10]. Furthermore, emerging research among Somali communities who have resettled in the USA and Europe demonstrate unexplained disparities in reproductive health outcomes [11–14]. Somali immigrants in the USA are a high-risk subpopulation possessing an increased risk for adverse pregnancy outcomes (cesarean deliveries associated with fetal distress, failed induction of labor, delivery beyond 42 weeks gestation, significant perineal lacerations, and poor neonatal outcomes), when compared to US-born Blacks and Whites [13]. Small and colleagues (2008) [14] conducted a meta-analysis of pregnancy outcomes among 10,431 Somalia-born women living in Australia, Belgium, Canada, Finland, Norway, and Sweden, who were compared to native-born women in the respective countries. While Somalia-born women were less likely to have preterm births (pooled OR 0.72, 95% CI 0.64–0.81) or infants with low birth weight (pooled OR 0.89, 95% CI 0.82–0.98), there were increased caesarean sections, particularly, in first births (pooled OR 1.41, 95% CI 1.25–1.59) and increased stillbirths (pooled OR 1.86, 95% CI 1.38–2.51). 

Consequently, Somali women appear to have unique needs during pregnancy and childbirth. However, given the high prevalence rates of the most severe form of FGC among Somali women, there is a dearth of evidence providing a direct and clear causal link between FGC and adverse reproductive health outcomes among migrant populations in Western societies [15, 16]. While some studies have exposed negative feelings and perceptions of discrimination in obstetrical settings as being associated with FGC and leading to difficult childbirth experiences, [17–20] other evidence suggests that various confounding factors, including clinical, cultural, linguistic, ethical, and personal beliefs, may contribute to a strong aversion to obstetrical interventions [15, 21, 22], consequently influencing suboptimal obstetric outcomes among this particular population in the West [13, 17, 19, 23–26]. As is the case for many immigrants, the Western biomedical approach to health care tends to be new to Somalis who also find the Western health care system overwhelming and difficult to comprehend and navigate [8]. For health care providers, caring for Somali women with FGC may present a challenge due to a host of sociocultural factors [17–19, 21, 22, 27, 28]. Hence discordant perspectives on health care expectations and recommended interventions between Somali women and their providers fuel patient-provider misunderstanding and miscommunication, which may have a negative impact on perceived quality of care and reproductive outcomes [21, 28]. Moreover, a growing body of evidence from different regions of the world, namely, Norway [20], Sweden [29], USA [30], UK [16, 31], and Spain [32], indicates the persistence of significant gaps in training providers, as well as gaps in general knowledge about caring for FGC-affected populations [33–35], even despite the availability of existing care protocols [16, 31]. This prompted an exploration of health care providers' perspectives surrounding reproductive care for Somali women. The specific aims of this study were to (1) explore providers' experiences, practices, and attitudes towards prenatal care and delivery of women with FGC, (2) examine providers' specific training on caring for women affected by FGC, and (3) determine the factors that influence the provision of quality care for Somali women with FGC. 

## 2. Methods

Development of the interview guide for this pilot study was informed by a review of existing literature as well as previous conversations with key informants from the Somali community. This was an exploratory questionnaire with open-ended questions arranged by themes and aimed at obtaining information about providers' knowledge, training, and practices related to the obstetrical management of circumcised women, providers' attitudes and perceptions of barriers (or facilitators) to the reproductive care of circumcised women, and providers' communication with circumcised patients. 

Investigators also developed a purposive sample of eligible participants by accessing the web sites of local hospitals and medical centers in areas where Somalis are known to access care and identified obstetricians at these hospitals or centers, where contact information of providers is usually available. In addition, based on the understanding that nurse midwives are often involved in the care of Somali women, a decision was made to interview licensed nurse midwives who cared independently for patients. Information about nurse midwives was obtained via the hospital and clinics' public websites or/and through referral from interviewed physicians. The study protocol and interview were approved by the Ohio State University Institutional Review Board. 

### 2.1. Sample and Data Collection

Between July 2007 and July 2008, an initial invitation letter was sent by e-mail to a list of OB/GYNs and nurse midwives in Columbus describing the purpose of the project and the expectation in time commitment should they decide to participate. Subjects were asked to reply to the letter and indicate their willingness to contribute to the project and to be contacted by the investigators by providing a telephone number and a time at which they could be reached. Each respondent received a follow-up phone call at which the meeting place and time were agreed upon; recruitment continued through snowball sampling. Thirty providers from six hospitals and clinics that care for a large volume of the city's Somali patients were contacted over a period of two months. Fourteen providers responded to our invitation and were recruited in the study. The returned letter signed by the provider was considered as an informed consent. Fourteen semistructured interviews (approximately 45 minutes in length) were conducted by a nurse midwife student in public health (Jalana N. Lazar) assisting the physician investigator (Michele P.-L. Shipp); these interviews took place over a period of two months in the spring of 2007. Each interviewee was offered a $15.00 restaurant gift card in appreciation for their contribution to the project (most of them declined). Interviews took place in hospitals' cafeteria, in hospitals' staff lounge, or in physicians' offices. Each interview was audiorecorded with the interviewee's explicit permission. All interviewees but one were White. There were 5 men and 9 women, including 9 obstetricians and one family practice physician with an OB concentration who was Somali, 3 nurse midwives, and 1 women's health nurse practitioner, ranging in age from 30 to 70, with self-reported 3–18 years of experience caring for Somali patients. 

### 2.2. Data Analysis

After each interview, the team reviewed the process, listened to the recording, shared impressions about the participant's responses, and made notes of salient perceptions about the interview. Throughout the data collection process, investigators continued to share their observations and insights about the interviews, informing the development of a coding theme. After interviews were completed, the recorded interviews were divided among the investigators (Michele P.-L. Shipp and Jalana N. Lazar) and transcribed verbatim. Each investigator then reviewed the transcripts individually, identifying frequent words or groups of words, making notes about perceived patterns or differences between respondents, proposing initial codes, and later reviewing them together for divergences or consensus. Subsequently, a qualitative analysis tool (QSR NVivo 9 software) [36] was used to facilitate a thorough content analysis of the collected text, to sort and arrange the transcribed information and to identify nodes and linkages. Codes were then arranged into subthemes and assessed for connections to prior research, allowing for discussion of areas of discrepancy and agreement. All identified subthemes were included in the results of this pilot study, with the exception of provider attitudes and perceptions towards the sexual experience of circumcised Somali women. This subtheme was not included in this paper as it was not identified by providers as a barrier to care. Those results were later published as an abstract [37]. 

## 3. Results

Our findings did not point directly to FGC as a primary barrier to the delivery of obstetrical care to Somali women. Instead, several themes were identified which elucidated barriers to the provision of quality care including: challenges in patient-provider communication, providers' frustration with perceived Somali women's resistance to obstetric interventions, providers' perception of mistrust by their Somali patients, and suboptimal provider training in the care and management of women with FGC.

### 3.1. Challenges in Patient-Provider Communication

Many participants reported communication difficulties as an important barrier in their care of circumcised Somali women. These difficulties appear to stem from the following factors. 

#### 3.1.1. Language

For some providers the Somali language posed a barrier as its non-Latin cadences, patterns, and sentence structure made providers completely reliant on interpreter services.

The language barrier, it's not like Spanish where you almost can pick it up because it does have some things that are the same in English language or fairly close or if you've spoken French or another European language where you can almost have some crossover. It sounds completely different, it has a totally different rhythm and doing that interview for your first visit is a long process and I've never over four years of residency learned any Somalian words and my Spanish, at least what I understand in Spanish has definitely improved over four years, and so it's just got a different rhythm to it, it's not like English at all and that is one of the hardest things to get over. (Female OB/GYN resident, 4th year (chief) caring for circumcised patients/Somalis)

I think I have sometimes some discomfort communicating because I'm not sure, and it's different from a romance language where even if you don't know the language you have some concept potentially of what's being said, whereas with Chinese or Somali you really have no idea, whether what I said in two sentences is really translatable into two words or vice versa or it takes half an hour to say the thing I just said in five minutes. (Female OB/GYN, 15 yrs. experience with circumcised patients/Somalis)

We don't speak their language and they only get enough of our language to feel we're denigrating their position although we aren't denigrating their position as much as they think we are. (Male OB/GYN, 18 yrs. experience)

We insist on an interpreter … even though they think they are getting along fine with English … we don't think they are and so we get an interpreter in that case. (Male OB/GYN, 11 yrs. experience)

#### 3.1.2. Interpretation

Several providers expressed concern about the objectivity and quality of interpretation services.

I think the Somali interpreters are, I don't mean to sound pejorative, not as reliable as some of the other interpreters, because they're filtering through the eyes of the tribe and what they need to do is to tell the patient what I tell them and I know that they don't, they modify it, because I've had others chime in and say “that's not what he said” so I'm suspicious that it's one of the problems we have in communicating, with the interpreter putting her own two cents in. (Male OB/GYN, 18 yrs. experience with Somalis/FGM)

I've actually had interpreters who don't exactly say what we say and sort of side with the family act as the patients advocate because they think we're trying to do something that's a problem. (Male OB/GYN, 10 yrs. experience)

I also think it's interesting because the Somali culture is so well knit here, most of the Somali patients know the interpreter on a personal level and then you wonder, you know I have no idea what they're saying cause I talked a lot and she said “blah blah” and that was it and I was like wow, I have no idea if that's right, I guess that is. (Female OB/GYN resident, 3 yrs. experience)

#### 3.1.3. Patient Autonomy

 Providers also cited communication difficulties that arose when patients insisted on having a family member or male partner interpret for them. This was especially frustrating to providers because they perceived that the woman was not communicating her own wishes. Providers had strong responses when their patients abdicated communication to their male partners and suggested that the male dominance of the communication deprived the patient of the autonomy they felt she should have in decisionmaking around her care.

I think for us, with women's health, it's the influence the men have over the women [that] is very difficult for us to understand, because again if a Somali woman is there and I'm consenting her for a C-section, I want her opinion, I don't want his opinion. They can talk amongst themselves but she needs to answer me and a lot of times they'll just look to the side and you have to talk to somebody else. (Female OB/GYN resident, 3 yrs. experience)

One of the things I find is that Somali women will just refuse to answer because they defer the question to their partner or their significant other. So if you have the discussion of the C-section, they say it is not up to me, it's up to my husband. Well actually, it is up to you. I have had that happen too, but not always, just sometimes. (Female OB/GYN resident, 3 yrs. experience)

… their male family members, who insist that they don't need an interpreter, that they or their family member speaks enough English. We usually try to insist pretty strongly that we get an interpreter because the message is never being communicated as well as we think. (Female OB/GYN, 6 yrs. experience)

It may be that the husband or the family member is doing the interpretation … it is impossible to know exactly who is making the decisions … she could be saying “no. I don't want that” and he is saying “we will have it” … and you don't know what she said … so you just take him at his word. (Male OB/GYN, 12 yrs. experience)

It's a culture that, to this day, I don't really understand; the role of the woman, the role of the pregnancy, the male domination … even for people who've been in America for a while, they still follow that, and how the male really dictates exactly what happens to the woman. We have interpreters come ad they'll even tell us that if we want to get a point across, we have to explain it to the husband first so that the wife will say ok and I don't understand why it's that way. (Male OB/GYN, 10 yrs. experience)

#### 3.1.4. Discomfort Discussing FGC with Patients

 Providers possessed varying levels of discomfort communicating with their patients about circumcision.

There are circumstances where a patient will ask questions and somehow open the door for me and then I feel more comfortable, but I don't really know how to communicate about that effectively … I guess my biggest fear is coming across really judgmental. I don't want to hurt a woman's feelings during an exam …. So I feel like that's a real deficit. (Nurse midwife, 6 yrs. experience)

It is really hard … my experience is that patients are pretty reticent about the procedures they have had … either the primary procedures or any corrective procedures … whether it is perceived in the communities that it is something not to be undone and they are trying to hush hush about it … I don't know … but my experience is that they are fairly reticent about it. (Male OB/GYN 12 yrs. experience)

I just try to be blunt about it and not make a big deal and hopefully that decreases their embarrassment about it. (Female OB/GYN 6 yrs. experience)

I've never had any problems with that. I realize it's their choice and it's just like the male mutilation we do, called circumcision. (Male OB/GYN, 18 yrs. experience)

I think the residents understand the medicine and the anatomy … but as to communicating … you know … medical necessity or certainly understanding the sociologic issues related to it … I don't think they are necessarily … especially the latter … well-grounded in that …. (Male OB/GYN, 12 yrs. experience)

### 3.2. Providers' Frustration with Perceived Somali Women's Resistance to Obstetric Interventions

All the providers interviewed were aware of Somali resistance to cesarean delivery and expressed strong conviction that Somali caesarean section rates were not appreciably higher than those of the general population. Many participants felt that the Somali perception that obstetrical interventions were higher than those of the general population was distorted by the fact that the rate of cesarean delivery in Somalia was certainly lower than in the USA. Participants seemed concerned at the possibility that Somalis perceived them as not having their patients' best interest at heart and were insulted by the Somali perception that providers performed cesarean sections because they were quicker, more convenient, or more lucrative.

Our sense of it is that they perceive we want to do C-sections because it is somehow faster, easier, or more financially rewarding for us, and that we don't want to wait for the vaginal delivery, we just want to push them to have a C-section. We sense that they think we are very quick to jump on C-sections, perhaps because of the language barrier or the cultural barrier. I don't feel that it is accurate. (Female OB/GYN, 6 yrs. experience)

We're not looking for C-sections to do. We try to do as many vaginal deliveries as we can. (Male OB/GYN, 18 yrs. experience)

they don't why know the doctors are doing the C-section … because of money, because they don't care … because it is easier … or there is some assumption that they are doing it more often for Somali women …. (Female family practice physician specializing in OB, 3 yrs. experience)

We definitely have a higher cesarean rate here then back in Somalia; I think it's just a US thing, medico-legal (Female OB/GYN, resident 3 yrs. experience)

Several participants alluded to incidents in which a Somali patient's refusal to have a cesarean delivery resulted in fetal death. Participants discussed these incidents in terms of cultural differences and values. Several talked about the Somali attitude that all decisions were in fact in the hands of God which they felt rendered the opinions and skills of medical providers irrelevant.

We said we thought she should have a C-section and they said, “No, it's Allah's will, it's Allah's will” and we watched the baby die and it was difficult. (Male OB/GYN, 18 yrs. experience)

They also have a lower expectation in terms of success with a pregnancy … we often hear … when we tell them: “You need a C-section or this baby is going to possibly die or have brain damage” … they say: “Whatever Allah wills”; so they are a lot more inclined to refuse C-section than the rest of our population …. (Male OB/GYN 10 yrs. experience)

Some participants spoke of the conflict they felt in the face of adverse outcomes that were a result of Somali patients' refusal to have a cesarean delivery … Many spoke of the difficulty of respecting the parents' cultural values when they felt these values endangered the baby, and they had been trained to save the baby whenever possible.

I certainly had an experience … that was very upsetting to me … here's what it is, she refused a C-section and we delivered a dead baby and in the US that just doesn't happen anymore, that's why we do so many C-sections cause we don't even let that possible opportunity occur, and for her that was her wish and it's very hard because you have to tell nursing staff and the med students because everyone's walking around in a huff “that's not good.” Well that's her belief. (Female OB/GYN resident, 3 yrs. experience)

We've seen patients in labor and delivery, on the heart rate monitor, where we all just watched the heart rate go away because they refused the C-section. (Female OB/GYN, 6 yrs. experience)

We've had some tragedies here with regard to infant death; which as a physician when you know that you can intervene in that situation and not have that outcome it's very, very difficult. (Female OB/GYN, 15 yrs. experience)

Many providers attributed the reluctance of Somalis to undergo cesarean delivery to the fact that it potentially limits their ability to have many children. Participants acknowledged this as a valid concern. However one provider expressed difficulty in understanding why a Somali patient would refuse an intervention that might result in a healthy baby in order to preserve the possibility of having more children later: “Cultural differences such as really not wanting the C-section I agree, because if she really wants to have seven babies I do not want to do seven C-sections because it gets more and more dangerous; but at the same time I'm not willing to sacrifice this child for six more whereas she might be willing to do that.” (Female OB/GYN resident, 3 yrs. experience). Overall providers conveyed that they knew these differences to be cultural and that they attempted to respect them in spite of not always understanding them.

The Somali resistance to induction of labor for postdates pregnancy and/or oligohydramnios was also a common theme. Although providers expressed admiration for the Somali desire to go into labor spontaneously, they felt the adamant stance against labor induction sometimes resulted in complications that might increase the chances of undergoing a cesarean delivery.

The other thing that Somali women are frequently very resistant to is the idea of inducing the labor. They want to wait for the labor to start spontaneously, which I agree with. I think the labor goes much nicer and is better for all involved if it starts spontaneously. Unfortunately, there are exceptions to that and sometimes we're concerned about the well-being of the mother or the baby and we have good reason to want to induce and it's a battle to convince Mom of that. (Female OB/GYN, 6 yrs. experience)

Just last week we had a problem where the fluid around the baby was gone and we were worried the baby was going to die, and we have to tell you that induction is the best thing and that probably labor isn't going to work because there's no fluid around the baby and we can try amnioinfusion and in fact the baby tolerated labor for half an hour and looked really bad and luckily they accepted the section, but if she'd had an induction two weeks earlier she might have had a different outcome, and it's hard to get that through to them. (Male OB/GYN, 18 yrs. experience)

I know that they're very resistant and it's hard, like with postdates. All the American women, when they're a day or two over they want us to induce them and the Somali women they're two weeks over and we're still nagging them, we'll schedule an induction and they don't show up. I think all that goes back to they're afraid that we're going to section them. I don't know how we can overcome that perception. (Nurse midwife, 4 yrs. experience)

But it is very difficult to talk to them … it does take a long time to convince them and encourage them to be induced … they show up two days later … then I have to talk to them to encourage them … sometimes they come forward … it may take two, three days … So … but I tell them about my personal experience … and then there are … misconceptions about why the induction … it is very linked to culture …. (Female family practice with OB training, 3 yrs. experience)

### 3.3. Providers' Perception of Mistrust by Their Somali Patients

Many providers stated that they perceived a sense of mistrust from their Somali patients and their spouses/partners and families, and they felt that this impacted their care. They felt this mistrust was a much greater barrier to providing quality care to Somali women than their circumcision status or other cultural factors. Providers were unsure about the origins of this mistrust and what could be done to mitigate it. However, a desire to understand and lessen the mistrust and misunderstanding was evident throughout the interviews.

I think they come in with some preconceived notions as well; that we're forcing health care on them, we're forcing tests on them that are unnecessary … so getting over that boundary, that's a barrier. (Female nurse practitioner, 5 yrs. experience)

The bigger problem is the belief in the community that we're somehow not acting in their best interest, especially when it comes to recommending induction of labor or cesarean section, or even limiting the number of children. (Female OB/GYN, 6 yrs. experience)

I think it's mistrust; I think they think we're trying to hurt them. I don't know why. I wish it wasn't there. It's very common and I don't understand it. (Male OB/GYN, 10 yrs. experience)

I think the trust issue is a big one. I would be very interested to understand their concerns about our care and this issue of C-sections. So I think this issue of trust is an enormous one and we should develop a better understanding of what that is. (Female OB/GYN, 15 yrs. experience)

### 3.4. Suboptimal Provider Training in the Care and Management of Women with FGC

Only one out of the 14 interviewed providers stated that she had received any type of formal training on the management of circumcised women prenatally and during labor and delivery. All the other study participants had learned on the job, either during residency training from a more senior resident or by being confronted with a circumcised patient at the time of delivery or during a pelvic exam. Some providers indicated that they would have appreciated more formal training while others felt it was unnecessary because they had become competent without training.

You sort of get dropped into it, I think we try to talk when there's a patient that we know is Somali whose having her 1st baby and is going to have a significant tear, I think we try to talk about how to manage that when we can. (Female OB/GYNB, resident 4 yrs. experience)

No formal training … we have such a large Somali population here in the clinic. You're taught early in your residency these are type I, type II, type III, here's what that entails, here's what we'll tell them, here's what we'll do for them. (Female OB/GYN resident, 3 yrs. experience)

I think the training is adequate now because of the numbers they see … it's really just a part of the training of episiotomy and episiotomy repair now … it is always part of that … as far as the procedure of de-infibulation … if they have cases in their GYN rotation where it is being done then it becomes part of their training … it is like any other surgery where it is not really a formalized piece of the training … because it is not that common of a surgery so …. (Male OB/GYN, 12 yrs. experience)

“I think they can muddle through it (circumcision management) …. I think we do OK with that … we do a pretty good job with our education in general … and in making those kind of decisions … ” (Male OB/GYN, 10 yrs. experience)

None of the clinical sites where study participants worked had formal protocols on the management of circumcised women. One participant mentioned that they had considered adopting a protocol to address requests for reinfibulation (re-approximation of the vulvar scar after vaginal delivery to resemble original circumcision); however, the protocol was never created.

There was an incident about 2 or 3 months ago where a patient of ours had delivered before out of state and the circ. was taken down during the delivery and then I don't know if it was a suture repair or if the tissue had just (come) together, so the way it healed, it looked as if it would need to be taken down again. With her delivery there was a big issue about that not being repaired the way that she wanted it to be done (post-delivery). So that kind of got some wheels turning in our department about maybe we should have a meeting to write things down. (Nurse midwife, 4 yrs. experience)

## 4. Discussion 

**Figure 1:**
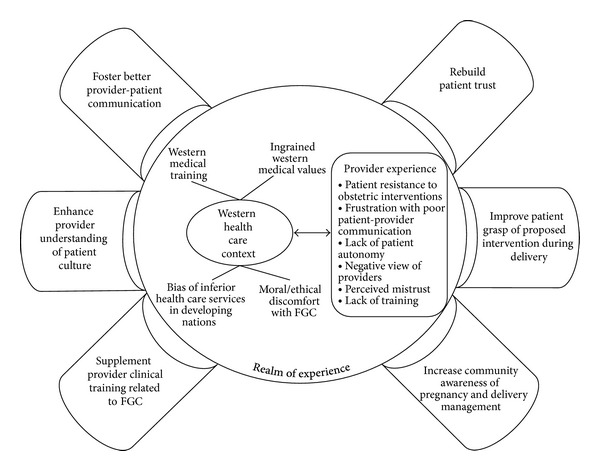
Mechanisms to improve the quality of care. Adapted from [48].

Although the results of our study do not reveal FGC to be a significant barrier to appropriate and effective care of pregnant Somali women as we had hypothesized, our findings point to other factors related to FGC that can indirectly influence the care of this group of patients. Based on these findings, we generated a conceptual model of barriers and facilitators that play a role in providing care to Somali women. As illustrated in Figure 1, our model takes into account the health care context, the providers' experience, and the behavioral mechanisms that could improve the quality of care.

The Western health care context is focused primarily on the body, on physical disease processes, and on illness, depends significantly on results of biological tests and evidence to guide treatment, and is usually aggressive in its pursuit of a cure and prevention of death. This approach tends to separate mind and body and often fails to consider other factors (psychosocial, spiritual, cultural, etc.) that might influence illness. On the other hand, a non-Western approach to health, such as is familiar to Somali immigrants, focuses on the person as a whole, where religion/spirituality, culture, traditions, and social network/support may play an important role in understanding and curing illness [38]. Moreover, Western medical and cultural attitudes towards FGC are generally negative and can provoke moral discomfort and ethical conflict for providers who care for circumcised women [28, 39]. Culture does matter and providers bring their own beliefs, biases, and cultural perspectives to their patient encounters. Understanding the cultural context around birth and the impact of FGC on delivery is crucial for providers to be able to create positive birth experiences for Somali women [15]. Lack of understanding or knowledge of patients' cultural background and beliefs has been found to be a significant barrier to appropriate and effective Western care delivery to ethnic minority groups, especially immigrants [40–42]. 

There is a notable intersection between patient-centered and culturally-competent care. Both models rely on the patient-provider partnership and the provider's recognition of the agency of the patient for effective health communication (the culture-centered approach) [42, 43]. In this study, providers' perceptions demonstrates a significant need for providers to feel valued as partners by Somali patients and to have their own input and competence recognized. There has been a limited examination of existing models of culturally competent care and the impact cross-cultural interactions have on provider attitudes and practices. In seeking to discover whether FGC presented a barrier to caring for Somali women, this study instead unearthed underlying mechanisms by which the existing Western health care context, patient mistrust, provider frustration, and cultural/value differences may erect barriers to effective patient-provider communication between Somali patients and their providers. 

Our finding of linguistic difficulties and doubt in the quality of interpretation services is also supported by evidence in the literature wherein providers did not deem interpreters sufficiently qualified to accurately convey provider information [39, 44]. Moreover, our findings that providers who care for circumcised women often experience conflicts between their own personal views on female empowerment, particularly in regards to male dominance over communication and decision making, have been substantiated by other studies [39, 45, 46]. The social, economic, and political structure of Somali society espouses a patriarchal community wherein gender inequality is pervasive and may pose a conflict with gendered norms in their host country of resettlement [47]. Furthermore, discordant views by Somali women and their providers may result in unmet needs and perceptions of diminished quality of care [28]. In our study, providers expressed concern that patients and their families feel they are being denigrated and at times may refuse an interpreter perceiving that their English fluency was sufficient. This has also been reported by Pavlish et al. where Somali women expressed frustration in the health care system's requirement that they receive a language interpreter, which they deemed condescending and time consuming [28]. 

In contrast, however, Binder et al. stress the importance of optimizing language congruence through the use of formally trained interpreters, as well as respectful, culturally competent, and professional encounters, which was deemed more important than cultural/ethnic or even gender concordance [44]. Recurrent themes that emerge in the literature are cultural/value clashes manifested in patients' reliance on religious beliefs and fatalistic attitudes [21, 39, 44], differing perceptions about FGC by patients and providers, a lack of antenatal discussion about FGC by providers [15], a feeling of being rushed to delivery, misperceptions about C-section [15], and a fear of dying from C-section [22]. These clashes played an important role in providers' attitudes towards Somalis' resistance to obstetrical interventions [15, 17, 22, 27]. Participant providers in our study identified these clashes as challenges to caring for their Somali patients, which may induce a generalized sense of frustration among providers. In agreement with the literature [21, 22], our results reveal provider frustration with their Somali patients' resistance to obstetric intervention, particularly labor induction and cesarean delivery, and further elucidate the factors which may explain the strong aversion to obstetric interventions, namely, poor patient/provider communication, provider frustration with the perceived lack of Somali women's autonomy in decision making, and sadness/indignation about Somalis' negative perceptions of provider intentions. 

Of note, our finding of providers' sense of powerlessness in averting unnecessary fetal death is in direct conflict with Somali women's fatalistic approach and religious coping mechanisms which further fueled providers' frustration. This is also congruent with the evidence-based literature wherein providers' personal and cultural values, as well as the contextual platform of Western medical training, may create conflict which may hinder the way they perceive their patients, communicate with, and provide care to them [21]. Moreover, it is possible that what may be deemed by providers as noncompliance and resistance to interventions from Somali women and their families, may in fact be a consequence of miscommunication and discordant misperceptions by both providers and patients [44]. 

All participants expressed deep concerns about a perceived lack of trust among Somali patients. The providers repeatedly identified a perceived mistrust among Somali patients as a significant barrier to providing quality obstetric care to this population. Other studies have correlated these conclusions [27, 49, 50] from the perspective of the Somali patient, but our findings highlight that providers perceive Somali patients' mistrust keenly although many seemed uncertain as to its etiology. Pavlish and colleagues [28] reveal that Somali women wished for a more personal relationship with their health care providers, felt rushed during encounters, and wanted longer consultations, whereas providers felt they spent too long with patients who need translator and try to take care of the problem as fast as they can. As one participant of the Pavlish study expressed, misunderstandings between well-intentioned providers and well-intentioned patients foster distrust and undermine relationships, and as a result, patients' health and well-being suffer. It is notable that our providers appeared to feel personally hurt by the perception of patient mistrust, and while they were able to appreciate Somali resistance to obstetric interventions in a wider historical cultural context, none applied this lens to the issue of trust. Improved culturally competent communication would likely improve this. 

It is interesting to note that our findings did not point directly to FGC itself as a primary barrier to the delivery of obstetrical care to Somali women but rather to the challenges in patient-provider communication, providers' frustration with perceived Somali women's resistance to obstetric interventions, providers' perception of mistrust by their Somali patients, and suboptimal provider training in the care and management of women with FGC. This is similar to the findings of Essén et al., 2011 [21], wherein providers did not describe FGC as a concern for Somali women's maternal care experience. However, we did not interpret this as a positive sign that knowledge about FGC had been effectively incorporated into the local health system. Thus, not perceiving FGC as a concern for the provision of quality care does not necessarily imply that adequate knowledge on the care of FGC-affected populations exists among health care providers. 

Studies in regions with significant number of immigrants from Somalia have found that often providers were not fully informed or prepared when encountering a circumcised patient and at times were uncertain as to how to best care for them in delivery. In a qualitative study conducted by Vangen and colleagues in Norway [20], health care professionals admitted to occasionally performing cesarean section in place of defibulation as they were uncertain how to perform a defibulation procedure. In the context of this study, one doctor reported that she was shocked after her first delivery with an infibulated woman, because she stated she did not recognize the anatomy. Other studies throughout North America and Europe have shown that a significant number of health care providers involved in the reproductive care of circumcised women have difficulty providing culturally competent care to these women and at times did not feel confident in their management of these patients. In a study by Ameresekere et al. in the USA [15], most women participants reported that their health care providers never discussed FGC or its potential for related complications of their delivery. Focus groups with circumcised women conducted by Thierfelder et al. (2005) in Switzerland [29] revealed a “striking lack of communication” about FGC between health care providers and their patients. Moreover, despite the availability of existing hospital protocols, and national guidelines, inappropriate management of FGC-affected patients, inadequate training of health care providers, and significant gaps in the provision of quality care still persist and have been attributed to language barriers, late initiation of prenatal care, provider discomfort, failure to identify FGC, and lack of a core training curriculum for providers [16, 31]. 

Despite expressing comfort in caring for circumcised women, a significant number of providers appear to be doing so without formal training, protocols and guidelines [29–32, 34]. Formal clinical protocols and guidance on overcoming communication barriers with circumcised women would result in more effective and satisfactory care [51, 52]. An educational tool has been developed by the American Congress of Obstetricians and Gynecologists (ACOG) in the form of a slide lecture kit on the clinical management of FGC [53]. However, unknown is the extent to which this educational resource is readily available and being actively utilized by residency training programs and providers across the USA, and how it is enhancing the quality of care and outcomes for women affected by this practice. A recent study by Jacoby and Smith [54] evaluated the effectiveness of an education program targeting midwives in the USA. This training included a didactic portion with a comprehensive review of ACOG guidelines and a hand-on training to build clinical management skills such as deinfibulation; the training was delivered to a group of midwives with the goal of improving their understanding of the unique needs of women with FGC and enhancing their ability to care for them. A pre- and posteducational survey demonstrated a significant increase in knowledge about FGC and improved confidence in being able to provide culturally competent, safe care to women with FGC. Similarly, Zenner and colleagues [16] examined the quality of care provided to circumcised women by obstetricians and midwives in a teaching hospital in the UK after the adoption of national and local protocols. Results of the study highlighted significant gaps in the practical application of these guidelines by health care providers and persistent deficits in the management of these women with FGC. These findings reinforce our assertion of the need for an increase in provider cultural competency in caring for FGC-affected populations, formal training to enhance knowledge on the care of circumcised patients, and an improvement in communication between patients and providers. Further research is necessary to inform the development of clinical training protocols and health policy recommendations to guide clinicians in the counseling and management of FGC, especially when ethical conflicts arise. 

 In order to improve trust and patient and provider communication on obstetrical care and aid in decision making, enhanced dialogue and anticipatory guidance are necessary early on and throughout antenatal care between providers, patients, and their families [15]. Somali women have clearly voiced their fear that undergoing a C-section will lead to death, or their body not returning to normal after surgery; that labor is rushed in the USA and they are pressured to delivery before they are ready; that patient-provider communication is important during labor and delivery; and that providers should discuss the implications of FGC on their care early in pregnancy [15]. Likewise, it is important to seek input from the Somali community on their views concerning obstetrical interventions, and cesarean delivery could provide an opportunity to enhance community health literacy, dispel fears, and reduce tensions with the health care system. Results from Somali focus groups and individual interviews conducted as a separate arm of a larger study may provide further insight into the nature of this distrust in the Somali community [55]. 

This study achieved thematic saturation in a number of areas including communication challenges, the lack of formal training for providers on the management of women with FGC, provider frustration with Somali attitudes towards obstetric intervention, and perceived mistrust. While the method of recruitment may have introduced a certain degree of selection bias, snowball sampling and purposive sampling have demonstrated reliability in the field. Efforts were made to mitigate this by recruiting obstetric care providers through a variety of communication channels. Some interview bias may have been introduced as both the interviewers and participants were health care providers, although concomitantly this may have contributed to enhanced disclosure among participants. Given the demands placed on the time of providers, interviews were occasionally rushed or interrupted which may have impacted the depth of some responses. Furthermore, the gender of the providers may have introduced some bias in patient-provider communication as well as influenced providers' perceptions of Somali women's receptivity to obstetrical care and interventions. Some studies have examined gender concordance in communication within multiethnic populations [44, 50]. While gender concordance is considered a priority and should be optimized for communication on highly sensitive gynecologic concerns such as FGC [50], having a competent and respectful provider has been shown to far outweigh a predilection toward sex distinction among providers [44]. 

Nonetheless, creating a true partnership between refugee communities and health care providers may lead to improved reproductive health outcomes and quality of care. Strategies to improve the quality of care for this population are to (1) Foster better patient-provider communication, (2) nurture patient trust, (3) enhance provider understanding of patient culture, (4) improve patient grasp of proposed intrapartum interventions, (5) supplement provider clinical training on FGC, and (6) increase community understanding of Western practices in pregnancy and delivery management. Future research should explore factors impacting communication between providers and their circumcised patients by examining the perceptions Somali women have of their reproductive care experiences and interactions with their providers. 

## 5. Conclusion

This paper presents unique perspectives of challenges providers face in caring for Somali refugee women with Female Genital Cutting (FGC). It also highlights communication challenges and cultural factors influencing Somali women's resistance to obstetrical interventions. We offer a model that illustrates various factors influencing patient/provider interactions and measures which may improve quality of care. This study adds to the growing body of evidence on the unique reproductive health care needs of Somali refugee women.
